# Celiac disease is associated with increased risk of deep vein thrombosis and hypotensive shock in patients admitted with acute pancreatitis

**DOI:** 10.1002/jgh3.70017

**Published:** 2024-08-24

**Authors:** Yazan Abboud, Vraj P Shah, Yi Jiang, Navya Pendyala, Kaveh Hajifathalian

**Affiliations:** ^1^ Department of Internal Medicine Rutgers New Jersey Medical School Newark New Jersey USA; ^2^ Karsh Division of Gastroenterology and Hepatology Cedars‐Sinai Medical Center Los Angeles California USA; ^3^ Rutgers New Jersey Medical School Newark New Jersey USA; ^4^ Division of Gastroenterology and Hepatology Rutgers New Jersey Medical School Newark New Jersey USA

**Keywords:** acute pancreatitis, celiac disease, National Inpatient Sample, outcomes

## Abstract

**Background and Aim:**

Celiac disease (CD) was shown to be associated with increased risk of developing acute pancreatitis (AP). There is a paucity of literature critically analyzing the association of CD with AP outcomes. We aimed to evaluate the impact of CD on outcomes and complications of AP in recent years.

**Methods:**

A population‐based analysis was performed using the National Inpatient Sample (NIS) between 2016 and 2019. Multivariable logistic regression was conducted to identify the independent impact of CD on AP outcomes while controlling for demographics and comorbidities and all patients refined diagnosis‐related groups (APR‐DRG) risk of severity subclass.

**Results:**

From 2016 to 2019, a total of 2 253 730 inpatients with AP were identified, of which 4640 (0.2%) had CD. On multivariable analysis, while controlling for demographics, comorbidities, and severity of illness, CD patients had significantly decreased odds for mortality (OR = 0.387), pseudocyst formation (OR = 0.786), sepsis (OR = 0.707), respiratory failure (OR = 0.806), acute kidney injury (AKI) (OR = 0.804), and myocardial infarction (OR = 0.217), (*P* < 0.05). However, CD patients were at significantly increased odds for deep vein thrombosis (DVT) (OR = 2.240) and hypotensive shock (OR = 1.718) (*P* < 0.05). Patients with CD had shorter lengths of stay by 0.4 days and lower total charges by $12 690.

**Conclusions:**

Our nationwide study evaluating AP outcomes in patients with CD suggests that patients with CD admitted for AP tend to have better mortality and several other outcomes compared to non‐CD patients. We also show that CD patients admitted for AP have a greater risk for DVT and hypotensive shock. Future studies are warranted to validate the revealed findings in CD patients admitted for AP.

## Graphical abstract

Nationwide inpatient analysis showing that patients with a history of celiac disease who are admitted for acute pancreatitis are at higher risk for developing deep vein thrombosis and hypotensive shock.

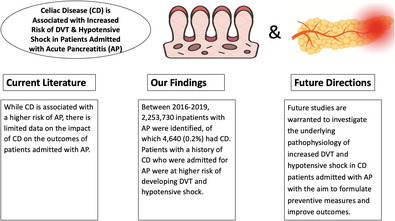

## Introduction

Celiac disease (CD) is a systemic chronic autoimmune disorder of the small intestines which occurs in genetically susceptible people exposed to a dietary protein diet and requires lifelong management.^1^ A systematic review and meta‐analysis of 50 studies showed that the incidence rates of CD have been steadily increasing over the last few decades.^2^ Previous data showed that patients with CD have a 2–3‐fold increased risk of acute pancreatitis (AP).^3^ With the tendency of developing AP in patients with CD, previous literature examined outcomes and showed that patients with a history of CD who are admitted for AP usually have better overall outcomes compared to non‐CD patients.^4^ For instance, a previous hospital‐based analysis showed that patients with CD admitted for AP tend to have better mortality.^4^ However, the aforementioned study only adjusted for a few variables in the analysis and did not evaluate many outcomes. Overall, there is a paucity of literature comprehensively analyzing the association of CD with AP outcomes and complications. Furthermore, there have been conflicting reports regarding venous thromboembolism risk in patients with CD.^5^, ^6^ Therefore, the aim of our study was to conduct a nationwide analysis evaluating the impact of CD on outcomes of AP in the inpatient setting.

## Methods

### Data collection

This is a retrospective population‐based cohort using the Healthcare Cost and Utilization Project (HCUP)'s National Inpatient Sample (NIS).^7^ The NIS is the largest inpatient all‐payer healthcare database in the United States, representing 95% of hospital discharges, covering more than 4000 nonfederal acute care hospitals in more than 40 states. The data include a principal diagnosis which is the primary discharge diagnosis, along with additional secondary diagnoses (up to 39) for a total of 40 diagnoses. All adult (aged 18 years or older) patients hospitalized with a principal diagnosis of AP between 2016 and 2019 were obtained from the NIS database and were classified based on the presence of CD.

### Definitions

Using the International Classification of Diseases, Tenth Revision (ICD‐10) diagnosis codes, the NIS was queried for inpatients with a diagnosis of AP using the following code (K85). ICD‐10 codes were also used to identify diagnoses of CD, comorbidities, and outcomes (Table S1, Supporting information). Patients' demographics included age, sex, ethnicity, median income by zip code, and insurance type. Comorbidities included cholelithiasis, choledocholithiasis, alcohol abuse/dependence, hypertriglyceridemia, bile duct obstruction, hypercalcemia, obesity, solid tumor status, inflammatory bowel disease, and autoimmune hepatitis. Outcomes included mortality, prolonged length of stay, future endoscopic retrograde pancreatography, pseudocyst formation, necrotizing pancreatitis, sepsis, respiratory failure, mechanical ventilation, acute kidney injury (AKI), ileus, hypotensive shock, myocardial infarction, stroke, portal vein thrombosis, deep vein thrombosis (DVT), parenteral nutrition, length of stay, and total charges. With the goal to cover most comorbidities, all patients refined diagnosis‐related groups (APR‐DRG) risk of severity subclass was utilized, which is a classification system that categorizes patients based on their severity of illness. The primary aim of the study was to evaluate mortality in patients with CD admitted for AP. Mortality was defined as death during the hospitalization (inpatient mortality), as per the NIS database. Secondary outcomes were to evaluate the rest of the aforementioned outcomes among those patients.

### Data analysis

Univariate analysis was conducted to compare continuous and categorical variables using *T*‐test and Fisher's exact test, respectively. A multivariable logistic regression module was conducted to identify the independent impact of CD on outcomes of AP while adjusting for demographics and comorbidities. A *P*‐value cutoff at 0.05 was utilized for statistical significance.

## Results

Between 2016 and 2019, a total of 2 253 730 inpatients with AP were identified, of which 4640 (0.2%) had a diagnosis of CD. Among those with CD (4640 patients), the mean age was 50.56 years, and the majority were females (69.4%), and of White race (85.8%). When compared to non‐CD patients, patients with CD tended to be younger, females, and of White race (all *P* < 0.001). The rates of cholelithiasis, choledocholithiasis, and Alcohol abuse/dependence were lower in AP patients with CD compared to non‐CD patients (all *P* < 0.001). All demographics and most comorbidities had significant differences between the two cohorts of CD and non‐CD patients (Table 1).

**Table 1:** Univariate analysis of demographic and comorbidity data of hospitalized patients with acute pancreatitis by celiac disease status

		Non‐celiac disease *n* = 2 249 094 (99.8%)	Celiac disease *n* = 4640 (0.2%)	*P*‐value
Age	Age, years (mean [SE])	52.68 [0.01]	50.56 [0.28]	<0.001
Sex	Male	52.1%	30.6%	<0.001
	Female	47.9%	69.4%	
Race	White	63.7%	85.8%	<0.001
	Black	15.8%	5.2%	
	Hispanic	14.0%	5.4%	
	Asian‐ z	2.5%	2.0%	
	Native American	0.9%	0.3%	
	Other	3.1%	1.2%	
Median household income by zip code	0–25th percentile	32.2%	16.4%	<0.001
	26th‐50th percentile	26.5%	28.2%	
	51st‐75th percentile	23.6%	27.6%	
	76th‐100th percentile	17.6%	27.8%	
Primary expected payer	Medicare	33.3%	31.3%	<0.001
	Medicaid	24.2%	15.7%	
	Private insurance	30.3%	45.7%	
	Self‐pay	8.4%	3.4%	
	No charge	0.7%	0.1%	
	Other	2.9%	3.8%	
Comorbidities	Cholelithiasis	20.3%	13.4%	<0.001
	Choledocholithiasis	8.6%	5.4%	<0.001
	Alcohol abuse/dependence	31.8%	18.8%	<0.001
	Hypertriglyceridemia	5.4%	5.2%	0.540
	Bile duct obstruction	2.7%	3.3%	0.010
	Hypercalcemia	1.0%	0.8%	0.070
	Obesity	17.8%	12.0%	<0.001
	Solid tumor	5.5%	4.6%	0.008
	Inflammatory bowel disease	1.4%	5.8%	<0.001
	Autoimmune hepatitis	0.1%	1.2%	<0.001

On multivariable analysis, while adjusting for demographics, comorbidities, and severity of illness using the APR‐DRG classification, patients with CD had decreased odds for mortality compared to non‐CD patients (odds ratio [OR] 0.387, *P* < 0.001). Furthermore, while also adjusting for demographics, comorbidities, and the APR‐DRG classification, patients with CD had decreased odds of pseudocyst formation (OR 0.786, *P* < 0.001), sepsis (OR 0.707, *P* < 0.001), respiratory failure (OR 0.806, *P* = 0.022), acute kidney injury (OR 0.804, *P* < 0.001), and myocardial infarction (OR 0.217, *P* < 0.001) (Table 2). However, patients with CD had significantly increased odds for developing DVT (OR 2.240, *P* < 0.001) and hypotensive shock (OR 1.718, *P* = 0.009). Lastly, patients with CD had a shorter length of stay by 0.4 days on average and lower total charges by $12 690.

**Table 2:** Multivariable analysis of comorbidity data of hospitalized patients with acute pancreatitis (AP)‐ associated with celiac disease

	Univariate analysis			Multivariable analysis† (association with celiac disease)		
Outcomes	Non‐celiac disease	Celiac disease	*P*‐value	Coefficient	95% Confidence interval	*P*‐value
	%			Odds ratio		
Mortality	45 095 (2.0%)	25 (0.5%)	<0.001	0.387	0.258–0.582	<0.001
Prolonged length of stay (≥14 days)^‡^	145 580 (6.5%)	225 (4.8%)	<0.001	0.945	0.810–1.102	0.470
Increased total charges (≥$202 990)^§^	95 465 (4.3%)	85 (1.8%)	<0.001	0.572	0.454–0.720	<0.001
ERCP^¶^	283 290 (12.6%)	505 (10.9%)	<0.001	0.978	0.867–1.102	0.711
Pseudocyst formation	125 625 (5.6%)	195 (4.2%)	<0.001	0.786	0.677–0.911	<0.001
Necrotizing pancreatitis	9055 (0.4%)	10 (0.2%)	0.044	0.601	0.322–1.121	0.109
Sepsis	266 075 (11.8%)	320 (6.9%)	<0.001	0.707	0.623–0.803	<0.001
Respiratory failure	167 250 (7.4%)	200 (4.3%)	<0.001	0.806	0.671–0.969	0.022
Mechanical ventilation	89 245 (4.0%)	75 (1.6%)	<0.001	0.581	0.450–0.750	<0.001
Acute kidney injury	401 865 (17.9%)	565 (12.2%)	<0.001	0.804	0.725–0.891	<0.001
Ileus	101 910 (4.5%)	165 (3.6%)	0.001	0.941	0.803–1.102	0.450
Hypotensive shock	11 195 (0.5%)	25 (0.5%)	0.692	1.718	1.147–2.575	0.009
Myocardial infarction	31 520 (1.4%)	10 (0.2%)	<0.001	0.217	0.116–0.406	<0.001
Stroke	8475 (0.4%)	20 (0.4%)	0.547	0.828	0.442–1.550	0.555
Deep vein thrombosis	12 765 (0.6%)	40 (0.9%)	0.008	2.240	1.629–3.080	<0.001
Parenteral nutrition	32 600 (1.4%)	55 (1.2%)	0.133	0.869	0.662–1.140	0.310

	Mean			Marginal values		
Length of stay (days)	5.44	4.78	<0.001	−0.399	−0.603 to −0.195	<0.001
Total charges ($)	61 325.08	44 245.53	<0.001	−12 690.365	−16 631.645 to −8749.085	<0.001

^†^ Multivariable regression analyses with covariables including demographic and comorbidity variables listed in Table 1 and all patients refined diagnosis‐related groups (APR‐DRG) risk of severity subclass.

^‡^ Prolonged length of stay defined as greater than one standard deviation above the mean length of stay.

^§^ Increased total charges defined as greater than one standard deviation above the mean total charges.

^¶^ ERCP (Endoscopic retrograde cholangiopancreatography).

## Discussion

Our nationwide study provides comprehensive data showing that CD patients tend to have better overall outcomes when admitted for AP compared to non‐CD patients. We also show that CD patients admitted for AP have a significantly increased risk of developing DVT and hypotensive shock despite adjusting for demographic and comorbidity variables, including solid tumor status which has been shown to be associated with an increased risk of DVT.^8^

It has been well documented in the literature that CD is significantly associated with an increased risk of developing AP. This association has been studied in both hospital populations,^4^ and large nationwide database populations.^1^ Numerous theories have been proposed to explain the underlying pathophysiology that may contribute to the increased risk of developing AP in patients with CD. For instance, CD is known to be associated with malnutrition which has been found to induce structural pancreatic changes leading to pancreatic acinar cell atrophy and fibrosis.^9^ These changes can impair pancreatic secretions potentially explaining the increased risk of developing AP in CD patients. Furthermore, the chronic inflammation seen in CD often leads to papillary inflammation, scarring, and stenosis which may sensitize patients to AP.^10^ There have also been data suggesting common immunomodulator pathways between CD and pancreatitis such as Th‐1 associated cytokines which are increased in both diseases.^11^, ^12^ Although the observed rates of DVT in our study are relatively low, these findings are consistent with previously published literature. We report that 0.6% of patients admitted with AP without CD and 0.9% with CD had acute DVT, compared to a previous NIS study that reported 0.4% of patients with AP developed acute DVT.^13^ While all of these theories might explain the increased risk of DVT in patients with CD, it is hard to draw any conclusions given the low percentage of patients who developed DVT. Future research is warranted to further investigate other etiologies and to better understand their clinical significance.

There is a gap in the literature regarding the effect of CD on AP outcomes. There has been one study done in the past to investigate differences in morbidity and mortality between AP patients with and without CD. Osagiede et al. examined data from 2007 to 2016 and demonstrated that ICU admission rate, shock, multiorgan failure, and mortality rates were better in AP patients comorbid with CD compared to non‐CD patients.^4^ However, our current study has several differences such as examining more recent data (2016–2019). Moreover, in contrast to the study by Osagiede et al. which utilized insurance carrier, hospital teaching status, and weekend admission as covariates in the multivariate regression, our study utilizes more covariates including demographic data and significant number of comorbidities with the goal to better estimate the individual impact of CD on AP regardless of other cofounders. Our study also shows that most patients with AP were younger, females, of White race, and less likely to have alcohol abuse/dependance, compatible with previous results seen in Explorys database.^14^ However, the previous study of Explorys examining outcomes of CD patients with AP showed higher risk of developing ileus, and no difference in pseudocyst formation, AKI, sepsis, or shock. This is contrary to our study in which CD patients had lower risk of pseudocyst formation, AKI, sepsis, a comparable risk of ileus, and a greater risk of hypotensive shock. The exact cause of the disparities in the results is unclear; however, it should be noted that the Explorys study evaluated the rates of these outcomes within 1 week to 1 month of having the AP episode, whereas our study reflected NIS data that include outcomes occurring within the given inpatient stay for AP. Moreover, the Explorys study used the Systematized Nomenclature of Medicine‐Clinical Terms (SNOMED‐CT) to identify patients with CD and AP compared to the ICD codes used in our analysis.

There are limited data explaining the disparities in AP outcomes between CD and non‐CD patients. One contributing factor could be that CD patients may be receiving additional medical surveillance for their CD which may also improve other clinical outcomes of AP. Some reports suggested that the reversal of intestinal damage in CD patients also restores exocrine pancreatic function.^15^ Moreover, the difference in etiology of AP between the CD and non‐CD patients has been well documented showing that CD patients usually have a higher risk of idiopathic AP. It also shows that AP in CD patients is often not associated with other AP etiologies such as alcohol, biliary, hypertriglyceridemia, and hypercalcemia, which can lead to more benign outcomes in CD patients given the association of those etiologies with worse outcomes.^4^ Lastly, despite adjusting for a significant number of comorbidities in our analysis, non‐CD patients were older and had higher rates of cholelithiasis, choledocholithiasis, alcohol abuse/dependence, and obesity compared to CD patients, which can still influence outcomes.

While AP patients with CD had lower risk of most clinical outcomes, they were at increased risk of DVT and hypotensive shock. Previous literature showed contradictory results about the risk of veinous thromboembolism (VTE) between the two cohorts, CD and non‐CD patients.^5^, ^6^ A cross‐sectional study did not show any increased risk of VTE among patients with CD.^5^ A case–control study in Denmark showed increased VTE risk only within 90 days of diagnosing CD.^16^ Our results are compatible with a recent systematic review and meta‐analysis of four studies showing a significantly increased risk of VTE in patients with CD.^17^ Contributing factors leading to increased risk of DVT in CD include malabsorption which can lead to vitamin deficiencies including Vitamin K and subsequently its derivatives protein C and S leading to increased risk of thrombosis.^18^ Another factor would be the chronic inflammation state in CD patients leading to the release of cytokines such as TNF‐alpha, which can also decrease the levels of proteins C and S.^19^ While CD individually confers a higher risk of DVT, the acute inflammation and production of cytokines such as TNF‐alpha, IL1, IL6, and IL10 in patients with AP have also been found to activate the coagulation cascade and inhibit the anticoagulation pathways.^20^ The increased isolated risk of DVT in both CD and AP patients can explain the increased odds of DVT in AP patients with CD as both at‐risk pathologies are present in these patients. Additionally, the etiology leading to an increased risk of hypotensive shock in CD patients is unclear and could be multifactorial due to the gastrointestinal bleeding seen in CD^21^ along with the usually lower systolic blood pressure seen in CD patients.^22^ Given that our reported rate of hypotensive shock (0.5%) is lower than other previously published studies, further analysis is required to better understand the clinical implications of these findings.^23^

Some of the strengths of our study include its large sample size, its evaluation of outcomes in recent years (2016–2019), and the conduction of a logistic regression model while adjusting for multiple demographic and comorbidity variables. Having said that, our study has several limitations. First, the retrospective nature of our current study hinders us from establishing any evaluation of causality between CD and AP. Furthermore, despite adjusting for multiple demographic and outcome characteristics, the retrospective design of our study limits our ability to account for all possible factors that can influence inpatient outcomes. As with any database study, we are limited by the accuracy of the data within the database. Specifically, there is a possibility of underreporting of diagnoses, such as that of CD. Moreover, we relied on ICD‐10 codes to identify patients with CD, without the ability to confirm via serological and histological data. Additionally, our analysis does not evaluate outcomes of AP in CD patients in outpatient settings. However, patients with AP are usually hospitalized and thus we believe that our analysis covers most AP cases in the United States.

Our nationwide study evaluating the outcomes of AP in patients with CD suggests that patients with CD who were admitted for AP tend to have better mortality and multiple other outcomes compared to non‐CD patients. However, we also show that CD patients admitted for AP tend to have greater risk for developing DVT and hypotensive shock. Future studies are warranted to investigate the revealed findings of a greater risk of DVT and hypotensive shock in CD patients admitted for AP and to possibly formulate preventive measures. Furthermore, future investigations are also warranted to further explore the risk of AP in patients with CD and the underlying pathophysiology.

## Supporting information


**Table S1.** ICD‐10 codes used to select cohort and identify complications and outcomes.
