# Supplementary material for: Celiac disease is associated with increased risk of deep vein thrombosis and hypotensive shock in patients admitted with acute pancreatitis
Source: JGH Open. 2024 Aug 24;8(8):e70017. doi: 10.1002/jgh3.70017 (PMC11344560; doi:10.1002/jgh3.70017)
Supplement: Supplementary file 1 — Table S1. ICD‐10 codes used to select cohort and identify complications and outcomes. [file JGH3-8-e70017-s001.docx]

**APPENDIX:**

**Supplemental Table 1: ICD-10 Codes Used to Select Cohort and Identify Complications and Outcomes**

| **Analysis Category** | **ICD-10 Code** | **Description** |
| --- | --- | --- |
| Acute Pancreatitis | K85.X | Acute pancreatitis |
| Celiac Disease | K90.0 | Celiac Disease |
| Comorbidities | K80.X | Cholelithiasis |
|  | K80.3, K80.4, K80.5, K80.6 | Choledocholithiasis |
|  | K70, F10, K860, K852 | Alcohol abuse/dependence |
|  | E781 | Hypertriglyceridemia |
|  | K831 | Bile Duct Obstruction |
|  | E8352 | Hypercalcemia |
|  | E660, E661, E662, E668, E669, Z683, Z684 | Obesity |
|  | C00-C96 | Solid Tumor |
|  | K50, K51 | Inflammatory Bowel Disease |
|  | K75.4 | Autoimmune Hepatitis |
| Outcomes | 0FJB8ZZ, 0FJD8ZZ, 0FHB8DZ, 0FHD8DZ, 0F798DZ, 0F798ZZ, 0F758ZZ, 0F758DZ, 0F768DZ, 0F768ZZ, 0F778ZZ, 0F778DZ, 0F788DZ, 0F788ZZ, 0F7D8ZZ, 0F7D8DZ, 0F7F8DZ, 0F7F8ZZ, 0F7C8ZZ, 0F7C8DZ, 0F9880Z, 0F988ZX, 0F988ZZ, 0F9980Z, 0F998ZX, 0F998ZZ, 0F9C80Z, 0F9C8ZX, 0F9C8ZZ, 0F9D80Z, 0F9D8ZX, 0F9D8ZZ, 0F9F80Z, 0F9F8ZX, 0F9F8ZZ, 0F9780Z, 0F978ZX, 0F978ZZ, 0F9680Z, 0F968ZX, 0F968ZZ, 0F958ZZ, 0F958ZX, 0F9580Z, 0FC58ZZ, 0FC68ZZ, 0FC78ZZ, 0FC88ZZ, 0FC98ZZ, 0FCC8ZZ, 0FCD8ZZ, 0FCF8ZZ, 0FF58ZZ, 0FF68ZZ, 0FF78ZZ, 0FF88ZZ, 0FF98ZZ, 0FFC8ZZ, 0FFD8ZZ, 0FFF8ZZ,  BF110ZZ, BF111ZZ, BF11YZZ, BF100ZZ, BF101ZZ, BF10YZZ, BF000ZZ, BF001ZZ, BF00YZZ | ERCP |
|  | K863 | Pseudocyst Formation |
|  | K8592 | Necrotizing Pancreatitis |
|  | R65, A40, A41 | Sepsis |
|  | J96 | Respiratory Failure |
|  | Z991, A19 | Mechanical Ventilation |
|  | N17 | Acute Kidney Injury |
|  | K56 | Ileus |
|  | R571 | Hypotensive Shock |
|  | I21 | Myocardial Infarction |
|  | I63 | Stroke |
|  | I824 | Deep Vein Thrombosis |
|  | 3E0336Z, 3E0436Z, 3E0636Z | Parenteral Nutrition |
